# Detection and Characterization of Nocardia from Patients Diagnosed as Tuberculosis in Egypt

**Published:** 2008-09

**Authors:** Zeinab H. Helal, Mazhar I. Khan, Mohamed Seif El-Din Ashour, Somaia A. Eissa

**Affiliations:** 1 *Department of Microbiology and Immunology, Faculty of Pharmacy, Al-Azhar University, Cairo, Egypt;*; 2 *Molecular Biology Laboratory, Department of Pathobiology and Veterinary Science, University of Connecticut, Storrs, Connecticut, USA;*; 3 *Center of Tuberculosis, Department of Microbiology and Immunology, Faculty of Medicine, Cairo University, Cairo, Egypt*

**Keywords:** Nocardia, *Mycobacterium tuberculosis*, real time PCR, 16S rRNA

## Abstract

Pulmonary tuberculosis and pulmonary nocardiosis are similar in most clinical symptoms and radiological manifestation. In the developing countries like Egypt where tuberculosis is very common, anti-tuberculosis drugs are started on basis of radiology and clinical symptoms. This study included 600 sputum specimens collected from 200 patients diagnosed as pulmonary tuberculosis from three chest hospitals in Egypt. IS6110 specific primer were selected for PCR to identify the *Mycobacterium tuberculosis* and hsp65 gene specific primers were used for PCR and sequencing for the identification of *Mycobacterium* and *Nocardial* species. The region of the gene coding for 16S rRNA in *Nocardia* species were selected as genus specific primer sequences for a PCR and Real Time PCR assays. Our result confirmed that four whole DNA samples, extracted from sputum specimen from the pulmonary tuberculosis patients on anti-tuberculosis treatment, were *Nocardia* species. Three of them matched (99% homology) with *Nocardia farcinica* (formerly *Nocardia asteroid* type V) and one match (83% homology) with *Nocardia pneumonia*. Molecular methods such as PCR and real-time PCR for identification of Nocardia are rapid and accurate methods. No cross-reactions were observed using Real Time PCR specific for Nocordia with other closely related genera.

## INTRODUCTION

Nocardia cause a variety of human infections including cutaneous, pulmonary and systemic nocardiosis. Nocardiosis is usually an opportunistic infection, and most commonly presents as pulmonary disease (1). Nocardia infection can occur by inhaling contaminated dust or via contamination of a wound with soil containing Nocardia (2). Nocardia infections have been seen in human and usually occur in patients with impaired local pulmonary defense or systemic immunosuppression (3, 4). People on chronic steroid therapy, those with cancer, organ or bone marrow transplantation, HIV/AIDS or tuberculosis are also at risk (4, 5, 6, 7).

Nocardial infection can be difficult to recognize, which leads to misdiagnosis and consequently underestimation of its incidence (1, 8, 9, 10, 11, 12). Pulmonary nocardiosis mimics pulmonary tuberculosis in both clinical symptoms, being chronic in nature and radiological characteristic making it difficult to differentiate from *M. tuberculosis* (6). Therefore, it is often wrongly treated with anti-tuberculosis drugs (4, 13). Molecular methods for identification such as16S rRNA PCR based assay, real-time PCR and sequencing offer a time saving for the diagnosis of Nocardial infection (14, 15).

In this study we have utilized four different molecular assays to specifically differentiate *Nocardia* spp from *Mycobacterium* spp from the DNA samples collected from the 200 patients diagnosed as tuberculosis at the three Chest Hospitals in Egypt.

## MATERIALS AND METHODS

### Collection and decontamination of clinical specimens

200 patients were included in this study after they were diagnosed as pulmonary tuberculosis by clinical symptoms, x ray, and microscopically by acid fast Ziehl-Neelsen staining method from the three specialized chest hospitals in Egypt. These patients were on anti-tuberculosis drugs for over two weeks to month. From these 200 patients, three successive days early morning sputum specimens were collected from each patient. Each three specimens were mixed and decontaminated by N-acetyl L-cystiene (NALC) sodium hydroxide (NaOH) method as described previously (16). Decontaminated specimens were concentrated by centrifugation at 3,000 × g for 20 minutes. All manipulations of specimens were done in the certified level II biosafety cabinet to contain the aerosols that potentially generated by adding reagents.

### Microbiological diagnosis

100 ul of centrifuged sediments were inoculated onto Lowenstein-Jensen (L-J) medium, and incubated for 8 weeks at 37°C with 10% CO_2_. Bacterial cultures were stained by Ziehl-Neelsen (Z-N) for the confirmation of acid fast bacelli.

### DNA extraction

The isolation of DNA was performed on the decontaminated specimens directly as well as from the suspended cultures using Qiagen DNA isolation kit (Qiagen, CA, USA) as per manufacture protocol at the Tuberculosis Center Laboratory, Faculty of Medicine, Cairo University, Cairo, Egypt. The concentration of DNA were determined by spectrophotometer using Bio Mate 5 (Thermo Spectronic, Rochester, NY) and stored at -20°C.

### PCR Specific for IS6110 gene

Two oligonucleotide primers (5'-GTGCGGATGGTCGCA GAGAT3') and (5'-CTCGATGCCCTCAC GGT TCA-3'), specific for IS6110 gene of *M. tuberculosis* were synthesized from the Biotechnology Facility at the University of Connecticut. These primers amplify 540 base pair DNA fragment specific for IS6110 gene (17). PCR reaction was performed in a 25 ul volume with master mix contained final concentration of 1.25U Taq DNA polymerase, 50 mM KCl, 30 mM Tris HCl, 1.5 mM Mg^+2^, 0.1% lgepal-CA630 and 200 uM of each dNTP (Eppendorf, NY, USA) with 100 pmol of primers with 100 ng of DNA template. Sterile de-ionized water was added to this mixture to bring the total volume to 25 ul. PCR was carried out in an Applied Biosystems thermal cycler (Applied Biosystems, CA, USA). The cycling protocol consisted of an initial denaturation at 94°C for 10 min, then 35 cycles that each consisted of denaturation at 94°C for I.5 min, annealing at 60°C for 1.5 min., and extension at 72°C for 110 seconds. The cycling was followed by final extension at 72°C for 30 min. A negative control was run with each test. The negative control did not contain template DNA and consisted of PCR master mix.

### PCR Specific for hsp65 gene

A 440-bp segment of 65-kDa heat shock protein (hsp65) for *Mycobacterium* and *Nocardia* species was amplified using (5’-ACCAACCATGGTGTGTCCAT-3’) and (5’-CTTGTCGAACCGCATACCCT-3’) oligonucleotide primers (18). PCR reaction was performed in a 25 ul volume with master mix contained final concentration of 1.25U Taq DNA polymerase, 50 mM KCl, 30 mM Tris HCl, 1.5 mM Mg^+2^, 0.1% lgepal-CA630 and 200uM of each dNTP (Eppendorf, NY, USA) with 100 pmol of primers with 100 ng of DNA template. The PCR amplification was carried out with an initial denaturation at 94°C for 1 min. followed by 45 cycles of denaturation at 94°C for I min, annealing at 60°C for 1 min., and extension at 72°C for 1 min. The cycling was followed by final extension at 72°C for 10 min.

### Sequencing of hsp65 PCR product fragment

The PCR products of hsp65 gene were purified by Qiagen PCR purification kit as manufacture instructions (Qiagen, CA, USA) and were submitted to sequencing (University of Connecticut Biotech facility center, CT, USA). The same primers used for PCR served for the sequencing of forward and reverse fragments. The sequence analysis was performed by blast analysis using DNA MAN software (Lynnon BioSoft, Vaudreuil, Quebec, Canada).

### PCR for 16S rRNA

16S ribosomal RNA (rRNA) gene segment specific for Nocardia species NG1 (5'-ACCGACCACAAGGGG-3') and NG2 (5'-GGTTGTAACCTCTTCGA-3') primers were used for PCR (15). PCR reaction was performed in a 25 ul volume with master mix contained final concentration of 1.25U Taq DNA polymerase, 50 mM KCl, 30 mM Tris HCl, 1.5 mM Mg^+2^, 0.1% lgepal-CA630 and 200 uM of each dNTP (Eppendorf, NY, USA) with 100 pmol of primers with 100 ng of DNA template. The thermal cycle consisted of initial denaturation at 94°C for 11 min. followed by 30 cycles of denaturation at 94°C for 1 min., annealing at 55°C for 20s, and extension at 72°C for 1 min. The cycling was followed by final extension incubation at 72°C for 10 min.

### Detection of amplified PCR Products

Agarose gel electrophoresis was used to detect PCR products. Ten micro liter volumes of PCR products were separated though a 1.5% agarose horizontal gel by electrophoresis at 84 volts. Gels were stained with ethidium bromide (0.5 ug/ml), and visualized by ultraviolet light and photographed.

### Real-Time PCR for Nocardia spp

The primers used for 16S rRNA PCR assay was also used for the Real-Time PCR using SYBR Green (9, 15, 19, 20). Applied Biosystems 7500 thermal cycler (Applied Biosystems, CA, USA) was used. The real time PCR reactions were carried out in 96 PCR plate in volume of 25ul containing 12.5ul from power PCR SYPR Green Master mix Kit (Applied Biosystems, CA, USA) 25 pmol from each specific primer, and 50 ng of DNA template. PCR amplification thermal cycle consisting of 50°C for 2 min and 95°C for 10 min followed by 40 cycles consisting of 95°C for 15 second and 60°C for 1 min. Negative control consist of master mix, water and *M. tuberculosis* H37Rv.

## RESULTS

### Microbiological and molecular diagnosis for TB

**Figure 1:**
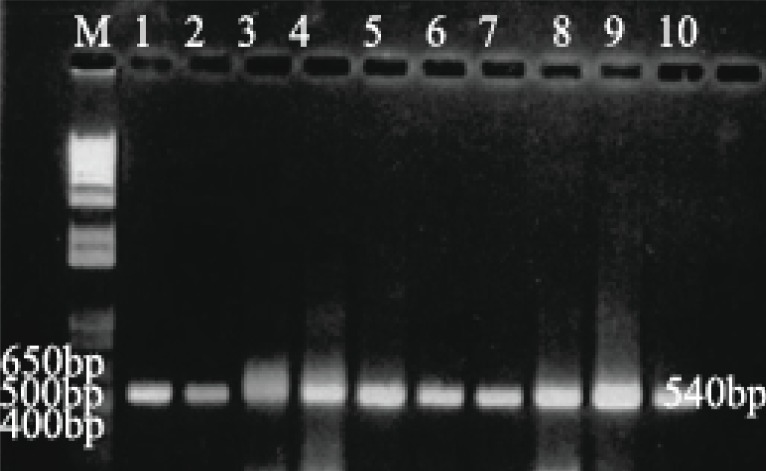
Agarose gel electrophoresis of positive 540 bp PCR products of IS6110 gene. Lane M, 1 kbp DNA ladder; Lane 1, *M. tuberculosis H37Rv*; Lanes 2-10, Random DNA samples from the 200 clinical sputum samples.

**Figure 2:**
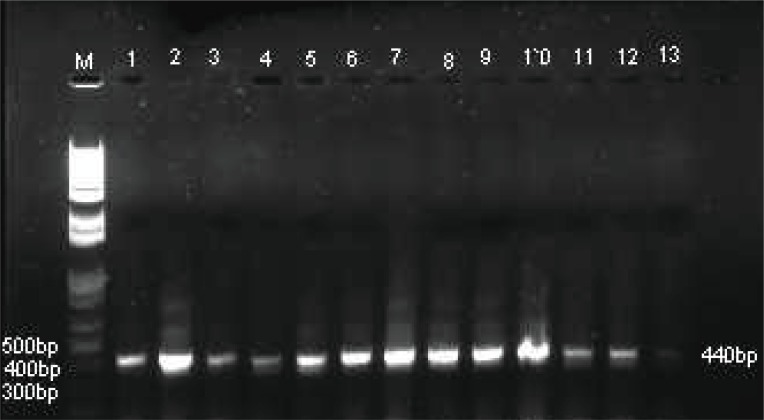
Agarose gel electrophoresis of positive 440bp PCR products of hsp65 gene. Lane M, 1 kbp DNA ladder; Lane 1, *M. tuberculosis*; Lanes 2-5, *Nocardia* species; Lanes 6-13, *Mycobacterium* species.

Microscopically using Z-N staining 151 decontaminated samples were positive by staining with Z-N stain, only fifty samples were positive by culture method after anti-tuberculosis treatment. However, 151 out of 200 DNA were positive for *Mycobacterium tuberculosis* using IS6110 specific PCR assay as shown in Table 1 and Figure 1. For remaining negative 49 samples with IS6110 PCR, 27 were tested positive for other *Mycobacterium* and *Nocardia* species using hsp65 specific PCR (Table 1, Figure 2). Where as 22 DNA samples were negative with hsp65 as well as IS6110 for *Mycobacterium* species.

**Table 1:** Results of diagnostic methods used on decontaminated sputum specimens

Methods	Z-N staining For Acid fast bacteria	L-J culture for *M. tuberculosis*	PCR of IS6110 specific for *M. tuberculosis*	PCR of hsp65 *Mycobacterium* and *Nocardia*	PCR of 16S-rRNA for *Nocardia* species only

Positive	151	50	151	27	4
Negative	49	150	49	22	23
Total	200	200	200	49	27

Z-N, Ziehl-Neelsen staining; L-J, Lowenstein Jensen medium.

### Detection of Nocardia by PCR and Real-Time PCR

**Figure 3:**
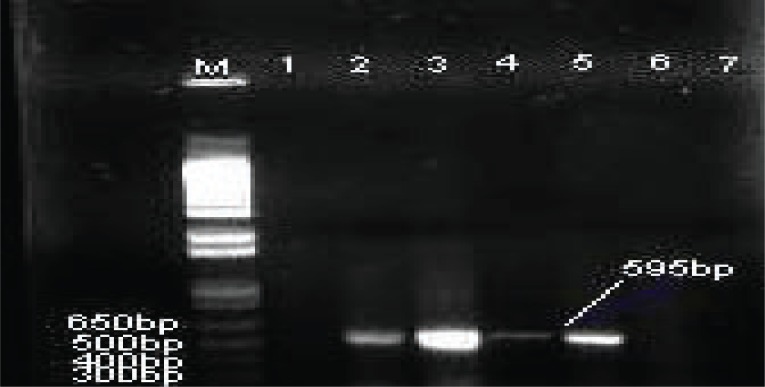
Agarose gel electrophoresis of PCR products of 16S rRNA gene by using primers NG1 and NG2 specific for detection of *Nocardia* species. Lane M, 1 kbp DNA ladder; Lane 1, *M. tuberculosis* H37Rv; Lanes 2-5, positive 16S rRNA 595 bp of *Nocardia species* from clinical samples; Lane 6, DNA from *M. tuberculosis* clinical isolates; Lane 7, DNA extracted from *Mycobacterium* chelonae.

PCR was used to determine the presence of Nocardia by using primers specific for Nocardia 16S rRNA on the positive 27 DNA samples that were *M. tuberculosis* negative but *Mycobacterium* or *Nocardia* spp. Positive. Four of these 27 DNA samples generated 595bp band specific for *Nocardia* species (Table 1, Figure 3). Moreover, the use of primers specific for Nocardia 16S rRNA, resulted in no DNA amplification was identified with the negative sample (H_2_O) or in the *M. tuberculosis* H37Rv, *Mycobacterium chelonae* or DNA positive with *M. tuberculosis* extracted from clinical isolates. (Table 1 and Figure 3).

**Figure 4:**
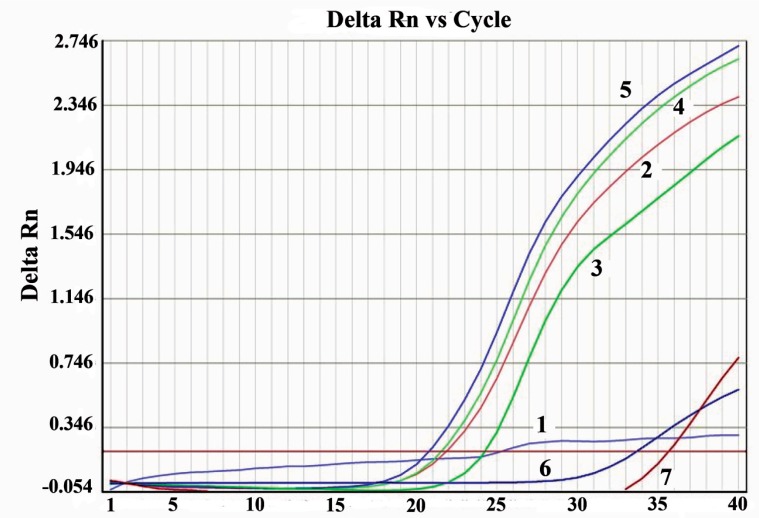
Detection of *Nocardia* species by real time PCR SYBR Green. Delta Rn, Display dye fluorescence as a function of cycle number; 1, negative (H_2_O); 2-5, positive with NG1 and NG2 primer specific for genus Nocardia; 6, *M. tuberculosis* H37Rv; 7, DNA from *M. tuberculosis*.

**Figure 5:**
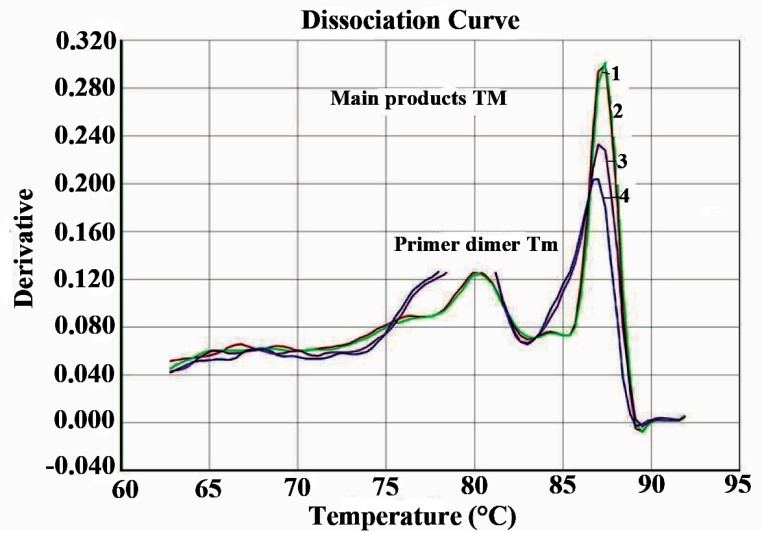
Melting curve analysis by real time PCR. Derivatives, displays a plot of the first derivative of the rate of change in fluorescence as a function of temperature; 1, negative (H_2_O); 2-5, positive with NG1 and NG2 primer specific for genus Nocardia; 6, *M. tuberculosis* H37Rv; 7, DNA from *M. tuberculosis*.

Real time PCR with SYBR Green was performed to further confirm their typical amplification and melting curves depicting Tm detection for Nocardia is shown in Figures 4 and 5. All four DNA samples detected as Nocardia spp. were positive after 15 cycles (Figure 4). Real time PCR specific for Nocardia 16S rRNA, resulted in no amplification with the negative sample (H_2_O) or in the *Mycobacterium tuberculosis* samples.

### Sequence analysis

Sequencing analysis using DNA MAN software on the sequence, of 27 amplified DNA products with hsp65 specific primers common to *Mycobacterium* or *Nocardia* species, was identified four amplicons as Nocardia species. Three of them were 99% match with *Nocardia farcinica* (formerly Nocardia asteroids type V) and one was 83 % match with *Nocardia pneumoniae*. While the sequence of the others 23 amplified DNA products with hsp65specific primers were mycobacterium species other than *M. tuberculosis*.

## DISCUSSION

Pulmonary nocardiosis is a sub acute or chronic pneumonia caused by aerobic actinomycetes of genus Nocardia. Most Nocardial infections are occurring in immunocompromised patients (21). Clinical manifestations of pulmonary nocardiosis are non-specific. It mimic’s pulmonary tuberculosis in both clinical symptoms and radiological characteristics (4, 6, 13). The chest radiographic manifestations are also pleomorphic and non-specific (22). In Egypt where tuberculosis is common, anti-tuberculosis drugs are started on based on clinical symptoms and radiological diagnosis.

In our study, 200 patients were initially diagnosed as pulmonary tuberculosis by the hospital based on clinical symptoms, chest x-ray and direct smear test by acid fast staining method. We found that 151/200 patients sputum samples were positive for *M. tuberculosis* by PCR analysis. There were 49 samples negative by the microscopical, culture or IS6110 PCR assay methods. Negative results may be due to the clinical specimens were collected after the anti-tuberculosis treatment started. In addition to, some anti-tuberculosis drug have early bactericidal activity which causes significant reduction in sputum colony forming unit count during the first few days of therapy (23). Moreover, L-J medium recover *M. tuberculosis* well, but is not as reliable for the recovery of other *Mycobacterium* species (24). Some of those negative samples may be hospital misdiagnosis. Of 49 negative, 27 were positive by PCR for hsp65. Four of these 27 were PCR positive specific 16S-rRNA primer specific nocarda. Sequence analysis of these four sample revealed that 3 out 4 were likely (99%) *Nocardia farcinica* (5, 6, 11) and one was most likely (83%) *Nocardia pneumonia* (25). Twenty two sputum samples that were remain negative for either mycobacteria or nocardia have resulted due to anti-tuberculosis treatment. Our results of 16S-rRNA PCR and real time PCR were consistence with the previous published results whether in the PCR amplified product (10, 15) or in the melting temperature curve obtained from real time PCR using SYBR green (9).

As shown in Table 2, the demographic and epidemiologic data obtained from medical records of 4 cases of Nocardiosis were under anti-tuberculosis treatment and they did not responded to the treatment. Specially there were two cases of them received anti-tuberculosis drug previously, hospitalized as relapse cases. Nocardia organism stain’s weakly or partially with Z-N staining on direct sputum smears labeled as positive has been described previously (4). We think that these 4 cases were identified as false positive for tuberculosis. On the other hand the sputum samples were decontaminated by NALC-NaOH method. The decontamination did not allow the growth of any other bacteria. Therefore, these four sputum samples of Nocardial patients had no growth onto Lowenstein-Jensen medium. The decontamination process and lack of special media has been linked to negative growth of Nocardia (4, 12). Follow up information on these patients is not available since these patients have left the hospital with reason unknown.

**Table 2:** Clinical data of four cases of Nocardia

	Case 1	Case 2	Case 3	Case 4

Age	59	56	35	51
Sex	male	male	male	male
Smoking	smoker	smoker	non smoker	smoker

**Hospital diagnosis**				
*a-Symptoms*				
Cough	+	+	+	+
Expectoration	+	+	+	+
Heamoptysis	+	-	-	+
Chest pain	-	+	-	-
Night fever	+	-	+	-
Night sweat	-	-	-	-
Weight loss	-	-	+	-
Loss of appetite	-	-	-	-
N.B	Chest trouble for one year before diagnosis		fatigue	
*b- Chest x-ray*	Tracheal shifted to right, raised upper vascular and right opacity	Left upper and middle zonal nonhomogenous opacity	Bilateral heterogeneous opacity	Mild zonal fibronodular opacity
*c-Medical history*				
COPD	-	+	+	-
Diabetes	DM	-	-	DM
Hypertension	-	-	-	-
Cancer	+	-	-	-
TB	Fresh case	Relapse case	Relapse case	Fresh case
*Microscopical* direct smear test for AFB	+	+	+	+
TB Treatment started	yes	yes	yes	yes

Heamoptysis, bloody cough; COPD, chronic obstructive pulmonary disease; DM, Diabetes mellitus; TB, tuberculosis; AFB, acid fast bacilli.

However, the present study highlights the need for detecting of pulmonary nocardiosis in Egypt especially among tuberculosis patients, due to the similarity of clinical and radiological examination between pulmonary nocardiosis and pulmonary tuberculosis especially with patients not responding to anti-tuberculosis drugs.
